# Methrotexate Treatment Inmunomodulates Abnormal Cytokine Expression by T CD4 Lymphocytes Present in DMARD-Naïve Rheumatoid Arthritis Patients

**DOI:** 10.3390/ijms21186847

**Published:** 2020-09-18

**Authors:** Jorge Monserrat Sanz, Cristina Bohórquez, Ana Maria Gómez, Atusa Movasat, Ana Pérez, Lucía Ruíz, David Diaz, Ana Isabel Sánchez, Fernando Albarrán, Ignacio Sanz, Melchor Álvarez-Mon

**Affiliations:** 1 Laboratory of Immune System Diseases, Department of Medicine, University Hospital “Príncipe de Asturias”, University of Alcalá, Alcalá de Henares, 28871 Madrid, Spain; alahoz1199@gmail.com (A.M.G.); david.diaz@uah.es (D.D.); 2 IRYCIS Unit, Instituto Ramón y Cajal de Investigación Sanitaria, 28034 Madrid, Spain; aperezalcala@yahoo.es; 3 Immune System Diseases-Rheumatology Service, Department of Medicine, University Hospital “Príncipe de Asturias”, University of Alcalá, Alcalá de Henares, 28805 Madrid, Spain; crisbohorquez@yahoo.es (C.B.); atusa_m@yahoo.es (A.M.); luciaruiz83@gmail.com (L.R.); aisatrio@gmail.com (A.I.S.); falbarranhdez@gmail.com (F.A.); 4 Division of Immunology and Rheumatology, Department of Medicine, Emory University, Atlanta, GA 30322, USA; ignacio.sanz@emory.edu

**Keywords:** naïve rheumatoid arthritis, CD4+ T-lymphocytes, methotrexate response, IFNγ, IL-17A, STAT expression

## Abstract

CD4^+^T-lymphocytes are relevant in the pathogenesis of rheumatoid arthritis (RA), however, their potential involvement in early RA remains elusive. Methotrexate (MTX) is a commonly used disease-modifying antirheumatic drug (DMARD), but its mechanism has not been fully established. In 47 new-onset DMARD-naïve RA patients, we investigated the pattern of IFNγ, IL-4 and IL-17A expression by naïve (T_N_), central (T_CM_), effector memory (T_EM_) and effector (T_E_) CD4^+^ subsets; their STAT-1, STAT-6 and STAT-3 transcription factors phosphorylation, and the circulating levels of IFNγ, IL-4 and IL-17. We also studied the RA patients after 3 and 6 months of MTX treatment and according their clinical response. CD4^+^T-lymphocyte subsets and cytokine expression were measured using flow cytometry. New-onset DMARD-naïve RA patients showed a significant expansion of IL-17A^+^, IFNγ^+^ and IL-17A^+^IFNγ^+^ CD4^+^T-lymphocyte subsets and increased intracellular STAT-1 and STAT-3 phosphorylation. Under basal conditions, nonresponder patients showed increased numbers of circulating IL-17A producing T_N_ and T_MC_ CD4^+^T-lymphocytes and IFNγ producing T_N_, T_CM_, T_EM_ CD4^+^T-lymphocytes with respect to responders. After 6 months, the numbers of CD4^+^IL-17A^+^T_N_ remained significantly increased in nonresponders. In conclusion, CD4^+^T-lymphocytes in new-onset DMARD-naïve RA patients show IL-17A and IFNγ abnormalities in T_N_, indicating their relevant role in early disease pathogenesis. Different patterns of CD4^+^ modulation are identified in MTX responders and nonresponders.

## 1. Introduction

The immune system plays a relevant role in the pathogenesis of rheumatoid arthritis (RA) [1,2]. However, the involvement of CD4^+^T-lymphocytes is only partially understood. CD4^+^T-lymphocytes form a regulatory and functionally diverse population of the immune system. This cell heterogeneity includes different patterns of cytokine secretion and stages of differentiation/activation [3,4,5,6]. CD4^+^T-lymphocytes subsets are characterized by their ability to produce cytokines such as IFNγ, IL-4 or IL-17A, and they are named Th1, Th2 and Th17, respectively [7,8]. Different signals are involved in promoting development, but the signal transducer and activator of transcription (STAT) family of proteins appears to be critical for the activation of the subset-characteristic transcription factors [9]. The binding of these STATs can activate lineage-specific enhancers associated with alternative cell fates [10]. STAT-1, STAT-6 and STAT-3 are recognized as essential for T-bet, Gata3 and RORc activation, which promote Th1, Th2 and Th17 cell differentiation, respectively. 

Based on their distinctive functional and phenotype patterns, CD4^+^T-lymphocytes are divided into CD4^+^ naïve (T_N_), central-memory (T_CM_), effector-memory (T_EM_) and effector (T_E_) T subsets [11]. CD4^+^T_N_ exhibits noneffector functions, while CD4^+^T_CM_ can rapidly proliferate and express multiple different effector molecules such as cytokines after being stimulated by antigens, and exhibit diminished activation requirements [11,12,13]. CD4^+^T_EM_ produce effector cytokines but have limited proliferative capacity, and CD4^+^T_E_ are at a final differentiation stage and share high levels of cytokine production [14]. The requirements for the activation, proliferation and survival of these subsets are different, as well as their capacity to enter lymphoid and inflamed nonlymphoid tissues [15].

RA patients show several abnormalities in circulating CD4^+^T-lymphocytes including an imbalance between circulating Th1, Th2 and Th17 subsets and abnormal serum levels of their hallmark IFNγ, IL-4 and IL-17A cytokines [16,17]. However, contradictory results have been published on the percentages of these T cell subsets and of the cytokine concentration in RA patients [18,19,20,21,22,23]. Several factors might be involved in this variability, including the clinical stage of the disease and the previous and active treatments. There is also evidence of an abnormal regulation of the expression and phosphorylation of the Th1, Th2 and Th17 inducers STAT-1, STAT-6 and STAT-3, respectively [24,25,26]. The treatment of RA has dramatically improved in recent decades by the introduction and use of methotrexate (MTX) [27]. MTX has become the most commonly used disease-modifying antirheumatic-drug (DMARD) in RA, but its mechanism of action remains elusive. Additionally, controversial effects have described the concerning results of MTX in the CD4^+^T-lymphocytes distribution and activity of the Th1, Th2 and Th17 subsets [16,19,22,23]. Thus, the analysis of Th1, Th2 and Th17 subsets in new-onset DMARD-naïve RA patients may clarify the role of these cells in the pathogenesis of the disease and the study of the immunomodulatory and clinical effects of MTX treatment may favor the understanding of the heterogeneity in the response to this DMARD. 

In this work, in a homogenous population of new-onset DMARD-naïve RA patients, we have investigated the pattern of IFNγ, IL-4 and IL-17A expression by T_N_ and T_CM,_ T_EM_ and T_E_ CD4^+^T-lymphocytes. We have also studied the expression and phosphorylation of the Th1, Th2 and Th17 transcriptional factors, STAT-1, STAT-6 and STAT-3, as well as the circulating levels of IFNγ, IL-4 and IL-17A. Furthermore, we have followed the patients during the first six month of MTX treatment and stratified them according to the clinical response attained.

## 2. Results

### 2.1. Patient Demographic Characteristics

Table 1 shows the baseline characteristics of the 47 new-onset DMARD-naïve RA patients who eventually became responders (*n* = 31) or nonresponders (*n* = 16) after six months of MTX treatment. No significant differences were observed in terms of age, sex and clinical variables examined between both groups of patients. We analyzed the evolution of C-reactive protein (CRP), disease activity score of 28 (DAS28) and the Health Assessment Questionnaire (HAQ) in both groups of patients at a 6-month follow-up. After six months of MTX treatment, the responders, however, showed a significant reduction in CRP from 16.12 ± 6.39 to 4.90 ± 2.31 mg/dl, in DAS28 from 3.62 ± 0.49 to 2.23 ± 0.41, and in HAQ from 0.76 ± 0.56 to 0.47 ± 0.26. The nonresponders also showed a significant reduction in CRP, from 16.57 ± 5.33 to 9.09 ± 4.18 mg/dL. The reductions in DAS28 from 3.69 ± 0.46 to 3.61 ± 0.25 and in HAQ from 0.78 ± 0.79 to 0.72 ± 0.65 were not statistically significant.

### 2.2. New-Onset DMARD-Naïve RA Patients Show An Expansion of CD4^+^IL-17A^+^ and CD4^+^IFNγ^+^ T-Lymphocytes

**Figure 1:**
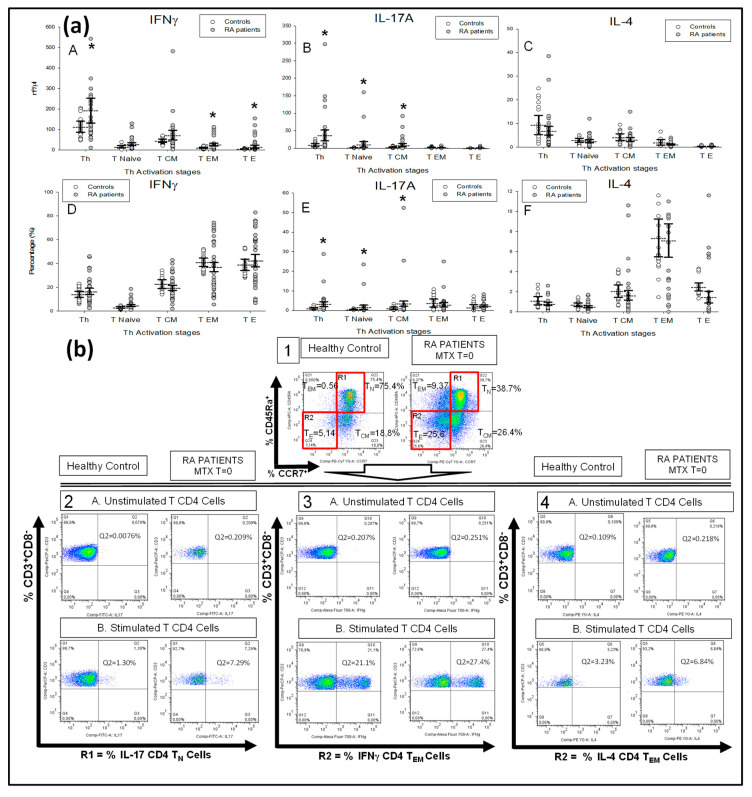
Intracellular IFNγ, IL-17A, IL-4 expression by the different activation/differentiation stages of T CD4^+^ lymphocytes from rheumatoid arthritis (RA) patients. Note: (**a**) Data represent numbers (nº/γl) (A, B and C panels) and percentages (%) (D, E and F panels) of total CD3^+^CD4^+^ (Th), and the T_Naïve_, T_CM_, T_EM_ and T_E_ CD4^+^ lymphocyte subsets that express intracellular IL-17A, IFNγ and IL-4 after in vitro phorbol-12-myristate-13-acetate (PMA) stimulation in disease-modifying antirheumatic drug (DMARD)-naïve RA patients (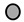) and healthy controls (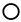). % (percentages) refers to total population of the indicated lymphocytes. All values are expressed as the mean cell numbers ± S.E.M. *, *p* < 0.05 for RA patients vs. healthy controls. (**b**) Panel 1. The first dot plots represent the selected gates and percentages of T_N_, T_CM_, T_EM_ and T_E_ CD4^+^ T-lymphocytes in two representative situations: a healthy control and RA patient at baseline before Methotrexate (MTX) treatment. Panel 2, 3 and 4. Dot plots represent the percentages of IL-17A, IFNγ and IL-4-producing CD4^+^ T cells in the presence and absence of PMA stimulation in the two representative cases described in panel 1. R1 and R2 represents the regions that include the T_Naïve_ and T_EM_ CD4^+^ lymphocyte subsets, respectively.

We investigated IL-17A, IFNγ and IL-4 expression by circulating CD4^+^T-lymphocyte subsets from 47 new-onset DMARD-naïve RA patients and 29 HCs (healthy controls) before starting MTX treatment and during the initial 6 months of treatment (Figure 1). There were no significant differences either in the number or in the percentage of circulating T-lymphocytes or CD4^+^T-lymphocytes between RA patients and HCs at baseline (T-lymphocytes: 2801.99 ± 380.15 vs. 2002.15 ± 347.12 cells/μL and 49.47 ± 2.52 vs. 52.88 ± 5.95%; CD4^+^ T-lymphocytes: 1131.60 ± 174.23 vs. 825.20 ± 79.84 cells/μL and 37.13 ± 2.17 vs. 40.29 ± 4.69%, respectively). 

Next, we investigated the IL-17A, IFNγ and IL-4 expression by PMA (phorbol-12-myristate-13-acetate)-activated CD4^+^T-lymphocytes from each individual. RA patients had significantly increased CD4^+^IL-17A^+^T-lymphocyte numbers with respect to HCs (Figure 1a, panel B). The percentage of CD4^+^IL-17A^+^ cells in the CD4^+^T-lymphocyte population was also increased in patients (Figure 1a, panel E). This CD4^+^IL-17A^+^ lymphocyte expansion could mainly be explained by a significant broadening of the CD4^+^IL-17A^+^T_N_ and CD4^+^IL-17A^+^T_CM_ lymphocytes (Figure 1a, panel E).

RA patients also showed a significant increment in the numbers of CD4^+^IFNγ^+^ cells with an expansion of the CD4^+^IFNγ^+^ T_EM_ and CD4^+^IFNγ^+^ T_E_ lymphocytes (Figure 1a, panel A). However, there were no significant differences in the percentages of IFNγ^+^-producing cells in the different CD4^+^T-lymphocyte subsets between patients and HCs (Figure 1a, panel D). The numbers or percentages of CD4^+^IL-4^+^T-lymphocytes were similar in both groups of subjects (Figure 1a, panel C and F). A representative dot plot of IL-17A, IFNγ and IL-4 expression by CD4^+^ T-lymphocytes is shown in Figure 1b.

**Figure 2:**
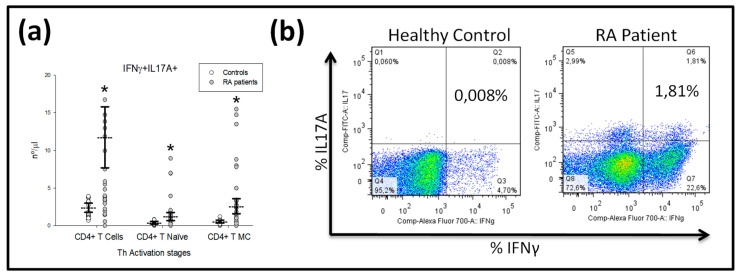
Intracellular IFNγ^+^IL-17A^+^ double-positive expression by CD3^+^, T_N_ and T_MC_ CD4^+^ lymphocytes in RA patients. Note: (**a**) Data represent numbers (nº/μL) of CD4^+^, T_Naïve_ and T_CM_ CD4^+^ lymphocytes in (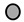) DMARD-naïve RA patients and (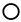) healthy controls. All values are expressed as the mean cell numbers ± S.E.M. *, *p* < 0.05 for RA patients vs. healthy controls. (**b**) Dot plots represent the percentages of IFNγ^+^IL-17A^+^ double-positive expression by CD4^+^ T-lymphocytes after in vitro PMA stimulation in two representative situations: a healthy control and a RA patient at baseline before MTX treatment.

Interestingly, the numbers of T_N_ and T_MC_ CD4^+^T-lymphocytes expressing both IL-17A and IFNγ were significantly increased in patients compared with HCs (Figure 2a).

**Figure 3:**
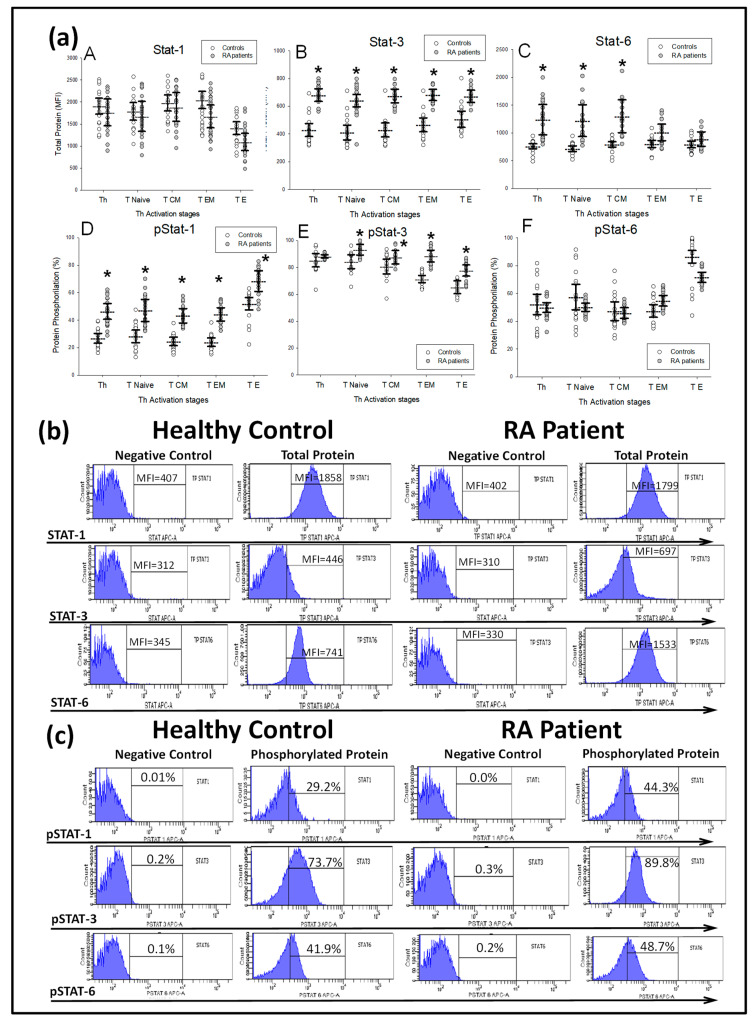
STAT-1, STAT-3 and STAT-6 phosphorylation and total protein expression by T CD4^+^ lymphocyte subsets from new-onset DMARD-naïve RA patients. Note: (**a**) Data represent the mean florescence intensity (MFI) and percentage (%) of the total and phosphorylated proteins, respectively, in CD3^+^CD4^+^, T_Naïve_, T_EM_, T_E_ and T_CM_ CD4^+^T-lymphocytes of (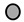) DMARD-naïve RA patients and (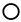) healthy controls. All values are expressed as the mean MFI or percentage ± S.E.M. *, *p* < 0.05 for RA patients vs. healthy controls. (**b**) Histograms represent the mean fluorescence intensity (MFI) of STAT-1, STAT-3 and STAT-6 total protein stimulated with IFNγ, IL-6 and IL-4, respectively, and their negative controls, in two representative cases: a healthy control and a RA patient at baseline before MTX treatment. (**c**) Histograms represent the percentages of STAT-1, STAT-3 and STAT-6 phosphorylation stimulated with IFNγ, IL-6 and IL-4, respectively, and their negative controls in two representative cases: a healthy control and an RA patient at baseline before MTX treatment.

Next, we investigated the expression and phosphorylation of STAT-1, STAT-3 and STAT-6 transcription factors in CD4^+^ T-lymphocytes from patients and HCs (Figure 3). Patients showed an increased percentage of phosphorylated STAT-1 and STAT-3 protein in the four different CD4^+^T-lymphocyte subsets analyzed with respect to HCs. Simultaneously, each CD4^+^T-lymphocyte subset from patients showed normal STAT-6 phosphorylation. Total STAT-3 protein was significantly increased in the four CD4^+^T-lymphocyte subsets from patients with respect to the HCs, while total STAT-6 protein was significantly increased in the CD4^+^ and T_N_ and T_CM_ subsets and total STAT-1 protein was normal.

**Figure 4:**
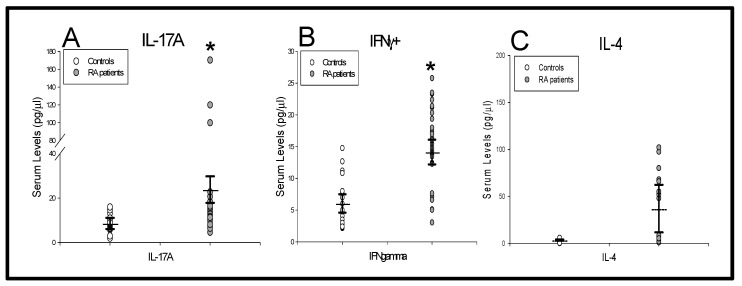
IL-17A, IFNγ and IL-4 serum levels in new-onset DMARD-naïve RA patients. Data represent the mean value serum levels of IL-17A, IFNγ and IL-4 in (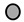) RA patients and (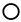) as healthy controls (panels **A**, **B** and **C**). All values are expressed as the mean serum levels ± S.E.M. *, *p* < 0.05 for RA patients vs. healthy controls.

Finally, we investigated the IL-17A, IFNγ and IL-4 serum levels from patients and HCs (Figure 4). We found that patients had significantly increased levels of IL-17A and IFNγ, but normal IL-4 concentrations.

### 2.3. Different Patterns of Distribution Of IL-17A, IFNγ And IL-4 CD4^+^ T-Lymphocytes Are Observed in MTX Responder and Nonresponder Ra Patients

In new-onset DMARD-naïve patients, we investigated the expression of IL-17A, IFNγ and IL-4 by circulating CD4^+^T-lymphocytes before and during the first 6 months of MTX treatment. We stratified the patients into two groups, which were defined according to the clinical response to MTX treatment attained after 6 months of treatment. There were 31 and 16 patients who met the criteria for responders and nonresponders to MTX, respectively. The patients were studied in parallel with 29 HCs. There were no significant differences in the number or frequency of circulating CD4^+^T-lymphocytes between MTX nonresponder and MTX responder RA patients and HCs at baseline (CD4^+^T cells: 821.99 ± 187.91 vs. 1101.54 ± 260.11 cells/μL and 33.07 ± 3.46 vs. 36.22 ± 2.73%, responder vs. nonresponder patients, respectively).

**Figure 5:**
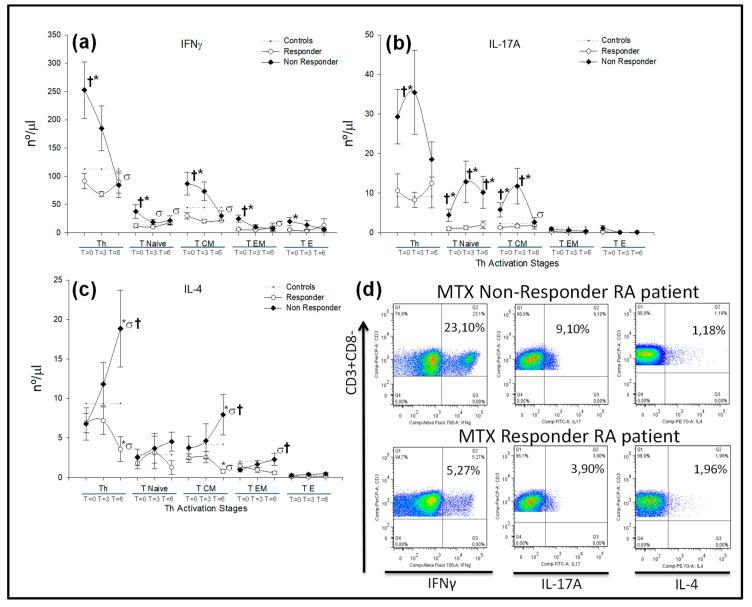
IFNγ, IL-4 and IL-17A-producing CD3^+^CD4^+^, T_naïve_, T_CM_, T_EM_ and T_E_ CD4^+^ T-lymphocyte numbers in RA patients according to MTX response. Note: (**a**–**c**) Data represent numbers (nº/μL) of IFNγ, IL-4 and IL-17A-producing CD3^+^CD4^+^, T_Naïve_, T_CM_, T_EM_ and T_E_ CD4^+^ T-lymphocytes according to the MTX response in (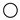) responder and (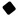) nonresponder RA patients. The dotted line represents the mean value recorded in healthy controls (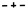). All values are expressed as the mean cell numbers (nº/μL) ± S.E.M. *, *p* < 0.05 for responder or nonresponder RA patients vs. healthy controls; †, *p* < 0.05 for responders vs. nonresponders, σ *p* < 0.05 for 6 months of follow-up time vs. baseline. (**d**) Dot plots represent the percentages of IFNγ^+^, IL-17A^+^ and IL-4^+^ expression by CD4^+^ T-lymphocytes after in vitro PMA stimulation in two representative situations: a nonresponder and a responder RA patient at baseline before MTX treatment.

Under basal conditions, MTX nonresponder patients showed a significantly increased number of CD4^+^IL-17A^+^T-lymphocytes with respect to responder patients, which could be explained by an expansion of the CD4^+^IL-17A^+^T_N_ and CD4^+^IL-17A^+^T_CM_ subsets (Figure 5b). After 6 months of treatment, there were no differences in the numbers of CD4^+^IL-17A^+^T-lymphocytes between nonresponder and responder RA patients, but the numbers of CD4^+^IL-17A^+^T_N_ remained significantly increased in nonresponders. During treatment, MTX responder patients did not show significant modifications in the number of CD4^+^IL-17A^+^T-lymphocytes.

Under basal conditions, the number of CD4^+^IFNγ^+^T-lymphocytes was significantly increased in MTX nonresponder patients with respect to responders, which was due to an increase in the CD4^+^IFNγ^+^T_N_, CD4^+^IFNγ^+^T_CM_ and CD4^+^IFNγ+T_EM_ subset numbers, which were significantly reduced after 6 months of MTX treatment. In MTX responder patients, there were no significant modifications of the CD4^+^IFNγ^+^T-lymphocytes numbers during the 6 months of treatment follow-up (Figure 5a).

There were no significant differences in CD4^+^IL-4^+^T-lymphocyte numbers between MTX responder and nonresponder patients under basal conditions. However, after 6 months of treatment, there was a significant increase in the number of CD4^+^IL-4^+^T-lymphocytes as well as in the CD4^+^IL-4^+^T_CM_ and CD4^+^IL-4^+^T_EM_ subsets in nonresponder patients. In contrast, MTX responder RA patients showed a significant reduction in numbers of CD4^+^IL-4^+^T-lymphocytes and CD4^+^IL-4^+^T_CM_ subsets (Figure 5c).

**Figure 6:**
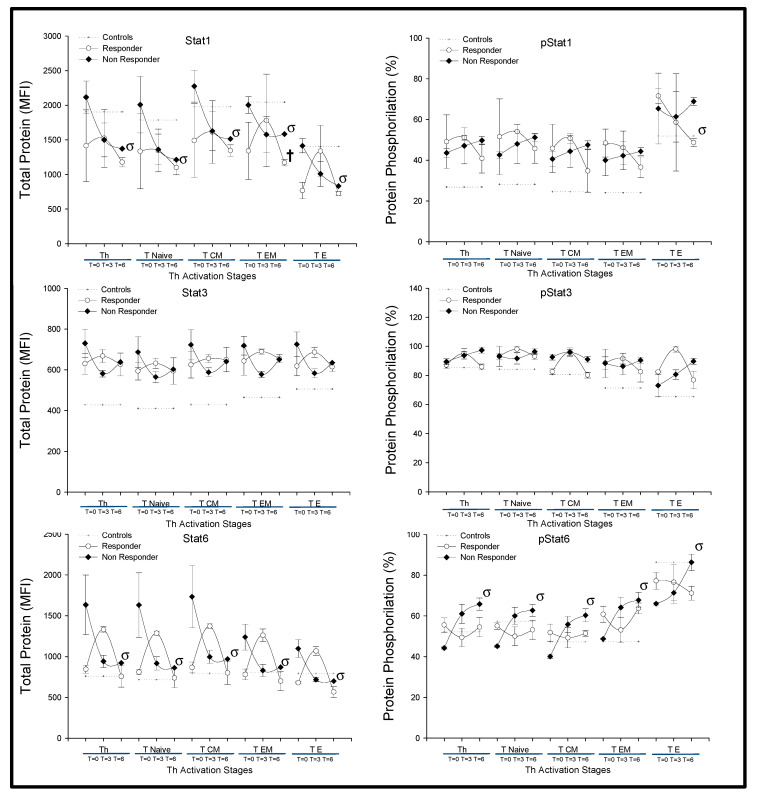
STAT-1, STAT-3 and STAT-6 phosphorylation in CD4^+^ T-lymphocytes of RA patients according to MTX response. Note: Data represent the mean florescence intensity (MFI) and percentage (%) of total and phosphorylated proteins, respectively, on CD3^+^CD4^+^, T_Naïve_, T_CM_, T_EM_, and T_E_ CD4^+^ T-lymphocytes according to the MTX response in (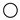) responder and (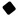) nonresponder RA patients. The dotted line represents the mean value recorded in healthy controls (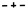). All values are expressed as the mean cell numbers ± S.E.M. †, *p* < 0.05 for responders vs. nonresponders, σ *p* < 0.05 for 6 months of follow-up time vs. baseline.

Next, we investigated the expression and phosphorylation of STAT-1, STAT-3 and STAT-6 transcription factors on CD4^+^T-lymphocytes from MTX responder and nonresponder patients. Under basal conditions and at 3 and 6 months, there were no significant differences in either the percentage of phosphorylation or in the total protein in the different CD4^+^T-lymphocytes between both groups of patients (Figure 6). However, there were significant differences in the total expression of STAT-1 and STAT-3 and STAT-6 phosphorylation by CD4^+^T-lymphocytes from MTX nonresponder patients between basal conditions and after six months of treatment.

**Figure 7:**
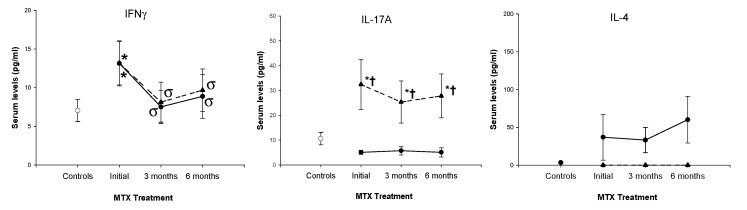
IFNγ, IL-4 and IL-17A serum levels in RA patients according to MTX response. Note: Data represent the mean value of serum levels of IFNγ, IL-4 and IL-17A in (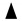) nonresponder, (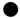) responder RA patients and (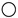) healthy controls. All values are expressed as the mean serum levels ± S.E.M. *, *p* < 0.05 for responders or nonresponders vs. healthy controls; †, *p* < 0.05 for responders vs. nonresponders, σ *p* < 0.05 for 3 or 6 months of follow-up time vs. baseline.

Finally, we also investigated the serum levels of IL-17A, IFNγ and IL-4 in RA patients before and during the initial 6 months of treatment. Significantly increased levels of IL-17A were detected in MTX nonresponders with respect to responders under basal conditions and during the six months of treatment follow-up. Bot, MTX responder and nonresponder patients showed similar basal levels and significant reductions in IFNγ levels during the 6 months of MTX treatment. IL-4 serum levels were significantly increased in MTX nonresponders with respect to MTX responders before and during the 6 months of treatment (Figure 7).

## 3. Discussions

In this paper, we have shown that new-onset DMARD-naïve RA patients have abnormally functioning circulating CD4^+^T-lymphocytes with an expansion of the CD4^+^IL-17A^+^, CD4^+^IFNγ^+^ and CD4^+^IL-17A^+^IFNγ^+^T subsets. This functional bias of the CD4^+^ T cell population is associated with increased intracellular STAT-1 and STAT-3 stimulation and increased circulating levels of IFNγ and IL-17A. Furthermore, the pattern of IL-17^+^, IFNγ^+^ and IL-4^+^ CD4^+^T-lymphocytes production detected in new-onset DMARD-naïve RA patients could be modified by MTX treatment, and two different behaviors were identified in responders and nonresponders.

CD4^+^T-lymphocytes play a critical role in the pathogenesis of RA [3,4,5,6,8,28]. Heterogeneous results, demonstrating increased, unchanged or reduced numbers and/or percentages of Th1, Th2 and Th17 CD4^+^T-lymphocyte subsets in the circulation of RA patients have been reported [19,20,21,22,23,29,30,31,32,33]. This variability may be explained by different nonmutually exclusive mechanisms, including disease duration, previous and active DMARD and immunosuppressor treatments, concomitant diseases, the genetic and epidemiological backgrounds of the patients, cohort size, and the methodologies used to record different immune system variables. To minimize these potential interferences with the mechanisms directly associated with RA pathophysiology, we focused herein on a clinically homogeneous population of new-onset DMARD-naïve patients. Our data revealed increased numbers of circulating CD4^+^IFNγ^+^ and CD4^+^IL-17A^+^T-lymphocytes in new-onset DMARD-naïve RA patients, but normal CD4^+^IL-4^+^T-lymphocytes. The frequency of Th17 cells was also increased, but the percentages of Th1 and Th2 lymphocytes were similar to those found in HCs. The differences in the results obtained using both methods of quantification indicate that the analyses of these CD4^+^T-lymphocytes subsets require the simultaneous study of numbers and percentages in RA patients. The numbers of circulating Th1, Th2 or Th17 cells appear to have special potential pathogenic relevance since they are a main source of IFNγ, IL-4 and IL-17A secretion [7,8]. In agreement with these cellular findings, the serum levels of IL-17A and IFNγ were increased, but those of IL-4 were normal in new-onset DMARD-naïve RA patients. Increased circulating IL-17A and IFNγ levels have been described in patients with early RA [34,35]. Interestingly, the numbers of circulating Th1, Th2 and Th17 cells showed differences under basal conditions and/or during MTX treatment between responder and nonresponder patients. Moreover, in agreement with a previous report, we found an expanded number of double IL-17A^+^IFNγ^+^CD4+T-lymphocytes in RA patients [23]. These different observations may contribute to understanding the established confusion concerning the normality or alteration of circulating Th1, Th2 and Th17 subsets in RA patients. Taken together, these findings improve knowledge of the involvement of CD4^+^T-lymphocytes in the early clinical stages of RA patients. Furthermore, this CD4^+^T-lymphocyte disturbance in RA patients cannot be ascribed to a single Th subset since both Th1 and Th17 were expanded with increased levels of circulating IFNγ and IL-17A cytokines. 

In addition to the pattern of cytokine production, CD4^+^T-lymphocytes are a heterogeneous population with different stages of differentiation/activation and patterns of circulation and tissue infiltration [11,12,13,14,15]. Interestingly, the number of CD4^+^T_N_ able to express IL-17A^+^ and IFNγ^+^IL-17A^+^ was increased in new-onset DMARD-naïve RA patients. These data suggest an abnormal bias of nonantigen activated CD4^+^T-lymphocytes from these patients toward IL-17A production, which is also observed in antigen-promoted T_CM_ CD4^+^T-lymphocytes. The relevance of the predisposition and acquired activation of CD4^+^T-lymphocytes to express cytokines is supported by the observation of the opposite results with respect to IFNγ production in naïve RA patients. The increasing numbers of IFNγ-producing CD4^+^T-lymphocytes were mainly focused in the CD4^+^T_EM_ and CD4^+^T_E_ subsets in new-onset DMARD-naïve RA patients. Different mechanisms might be involved in these different functional findings, including the intrinsic/genetic characteristics of the patients, the activating microenvironment and preferential extra-vascular tissue migration, such as the inflamed joints in naïve RA patients. It has been proposed that the CD14^+high^CD16^+^ monocyte subset participates in the expansion of Th17 T-lymphocytes in RA patients [36,37]. There is also evidence supporting the relevance of the cytokine microenvironment in the differentiation of naïve T-lymphocytes into Th1, Th2 and Th17 subsets [7]. Furthermore, precursors such as CD4^+^CD161^+^T-lymphocytes may differentiate into either Th1 or TH17 lymphocytes based on the presence of IL-1γ and IL-23 or TGFγ [7]. It is possible that this plasticity might be involved in the observed expansion of CD4^+^T_N_ able to express IL-17A^+^ and IFNγ^+^IL-17A^+^ in new-onset DMARD-naïve patients with RA. In contrast, the selective overexpansion of IFNγ in CD4^+^T_EM_ and CD4^+^T_E_ lymphocytes suggests the occurrence of antigen stimulation in the IL-12 microenvironment.

The relevance of the different signals driving Th lymphocyte activation appears to be critical because the percentages of phosphorylation of the transcription factors STAT-1 and STAT-3 were increased in the four different CD4 differentiation/activation stages in the DMARD-naïve RA patients. It is possible to suggest that early RA is associated with an intrinsic CD4^+^IL-17A^+^T_N_ differentiation. However, antigen pressure and cytokines favor Th1 differentiation with a predominance of CD4^+^T_EM_ and CD4^+^T_E_ lymphocyte activation. In addition, it has been proposed that Th17 cells are unstable and easily shift toward Th1 cells, named “non-classic Th1 cells”, and they have been found in early RA with relevant pathogenic activity [34]. In cord blood or spondyloarthropathies, abnormal Th1 and Th17 differentiation have been postulated in response to IL-1β and IL-23 [38,39]. Our data showed the involvement of both Th1 and Th2 subsets in early RA patients; however, their pathogenic role remains to be elucidated.

Our data revealed a heterogeneous function of CD4^+^T-lymphocytes in the early stages of RA. Analysis of the basal characteristics of new-onset DMARD-naïve patients who did not achieve a clinical response to MTX showed a significant expansion of CD4^+^IFNγ^+^ and CD4^+^IL-17A^+^ T_N_ and T_CM_ and CD4^+^IFNγ^+^ T_EM_ and T_E_ lymphocytes with respect to those circulating in responders. Interestingly, during the 6 months of follow-up, MTX nonresponders maintained increased numbers of CD4^+^IL-17A^+^T_N_ cells. Nevertheless, a normalization of CD4^+^IFNγ^+^ cell subset numbers was observed with a concomitant increase in CD4^+^IL-4^+^T_CM_ and CD4^+^IL-4^+^T_EM_ cells. In addition, the significantly increased levels of circulating IL-17A persisted, but those of IFNγ were normalized, during the 6 months of MTX treatment in nonresponders. Thus, it is possible to suggest that the persistence of Th17 polarization during MTX treatment is associated with a defective response to treatment with this drug in early RA patients. The relevance of this Th17 differentiation is also supported by the normal numbers of CD4^+^IFNγ^+^, CD4^+^IL-17A^+^ and CD4^+^IL-4^+^ subsets in MTX responder patients. These results support the idea that these Th17 might be important in RA pathogenesis and in the response to immunomodulator treatments. There were no significant differences in the levels and phosphorylation of the STAT-1, STAT-3 and STAT-6 proteins in the different CD4^+^T-lymphocytes between responders and nonresponders. Interestingly, the different behaviors of the CD4^+^T-lymphocytes compartment in both groups of patients cannot be ascribed to different disease activities before starting MTX treatment.

The precise mechanism of action of MTX in RA patients remains obscure [40]. It may act by decreasing cell proliferation, enhancing the rate of apoptosis, increasing endogenous adenosine concentrations, or altering cytokine production [41,42]. However, MTX is not a general antiproliferative drug; indeed, it induces apoptosis only in highly activated immune system cells [42,43]. The present data indicate that MTX causes different regulatory effects on CD4^+^T-lymphocytes in responders and nonresponders. This different CD4^+^T-lymphocyte modulation cannot be ascribed to a differential effect on the levels and phosphorylation of STAT-1, STAT-3 and STAT-6 proteins. The absence of a clinical response to MTX does not, however, rule out a biological effect of the drug on patient CD4^+^T-lymphocytes. Furthermore, the progression of uncontrolled diseases may be related to the expansion of CD4^+^IL-17A^+^T_N_ lymphocytes. Determining whether this is the case is impossible since it would be unethical to maintain patients with active RA without treatment. These results support the knowledge of the relevance of an early immunomodulation in a subset of new-onset RA patients. Future works have to investigate the potential value of CD4^+^T-lymphocytes parameters as biomarkers in new-onset DMARD-naïve RA patients.

## 4. Materials and Methods

### 4.1. Inclusion and Exclusion Criteria

The study subjects included 47 Caucasian patients with ACR/EULAR (American College of Rheumatology/The European League Against Rheumatism) 2010 classification criteria for RA [44]. Patients were studied in parallel with 29 healthy sex-, age-, ethnicity-matched controls. The patients were followed at the Rheumatology Service, Hospital Príncipe de Asturias, Alcala University, in Spain. All patients provided their informed consent to be included. The study was properly approved by the hospital’s clinical/ethics committee: “Ethical committee for clinical research of the Hospital Principe de Asturias” (research proyect 1/2006, code P1-11-02433, committee approval date 26/1/2006).

Inclusion criteria:

Patients with new-onset RA (disease duration < 3-months), previously untreated with DMARDs and a disease activity score of 28 (DAS28) according to EULAR criteria, were evaluated for inclusion in the study [44].

Exclusion criteria:

To have (1) severe cardiovascular disease, (2) hypercholesterolemia or diabetes mellitus, hematopoietic, (3) lung, hepatic or renal disorders, (4) acute or chronic bacterial or viral infections, (5) other autoimmune diseases, (6) treatment with steroids, immunosuppressors or other drugs that would have interacted with the immune system in the previous 6 months, (7) possible pregnancy/lactation during the study period, and (8) simultaneous malignancy or congenital immunodeficiency.

### 4.2. Study Protocol

All patients were treated weekly for 6 months with 15 mg MTX (orally) plus 5 mg folic acid. The MTX dose was adjusted by increments of 5 to 25–30 mg weekly until the disease response criteria were met. Patients were advised to take 5 mg of Prednisone (orally) daily and a non-teroidal anti-inflammatory drug at fixed doses during the study. All patients were monitored monthly for clinical and analytical tolerance to MTX treatment and at 3 and 6 months to assess the clinical response and to undertake immunological studies. Disease activity was determined by the DAS28 score according to EULAR criteria and using a validated Spanish version of the Health Assessment Questionnaire (HAQ) [45]. The clinical response of the patients to MTX was defined according to EULAR criteria for RA [46], classifying patients as responders or nonresponders. The responder group included those patients with an actual DAS28 < 3.2 score, plus a DAS28 score decrease by at least 1.2 with respect to the initial value, after 6 months of MTX treatment.

Three peripheral blood samples were obtained from each patient by antecubital venipuncture at baseline (before starting MTX treatment) and at 3 and 6 months after starting MTX treatment.

### 4.3. Clinical Laboratory Assays

C-reactive prtein (CRP) y Rheumatoid factor (FR) was determined by an inmmunoturbidimetry assay in an Atellica Solution^®^ CH (Siemens Healthineers, Erlangen, Germany), with 0–5 mg/L being the normal range for CRP and 3.5–90 IU/mL for RF. Anti-CCP was determined by a fluoroenzimo-immunoassay (Inmunocap 250, Thermofisher Scientific, Waltham, MA, USA) with a normal range under IU/mL.

### 4.4. Isolation of Peripheral Blood Mononuclear Cells

Peripheral blood mononuclear cells (PBMCs) were obtained from heparinized venous blood and were separated by Ficoll-Hypaque (Lymphoprep^TM^, Axis-Shield, Oslo, Norway) gradient centrifugation [47]. They were then resuspended in RPMI-1640 with 10% heat-inactivated fetal calf serum (Gibco, Life Technologies Limited, Renfrew, UK), 25mM HEPES and 1% penicillin-streptomycin (Biowhittaker, Lonza, Barcelona, Spain). Cell enumeration was performed as previously described [48]. The PBMCs of each patients or control were adjusted to 1 10^6^ cells/mL prior to antibody staining.

The cell number counts of lymphocytes subsets were calculated by the percentage of each subpopulation in the PBMCs determined by flow cytometry multiplied by the total number of lymphocytes per microliter obtained by a complete blood count from a conventional hemogram measured by Beckman Coulter, Inc (Brea, CA, USA).

### 4.5. In Vitro Culture

The spontaneous and stimulated T-lymphocyte subset expression of IFNγ, IL-17A and IL-4 was assessed by in vitro intracytoplasmic staining in the presence of 2mM monensin. The PBMCs were stimulated with 50 ng/mL phorbol-12-myristate-13-acetate (PMA, Sigma-Aldrich, MerkMillipore, Boston, MA, USA) plus 1μg/mL ionomycin (Calbiochem, MerkMillipore, Boston, MA, USA) for 6 h. Spontaneous cytokine expression was determined in parallel cultures in the absence of exogenous stimuli.

### 4.6. Surface and Intracellular Lymphocyte Staining

T-lymphocytes were studied in PBMCs by nine-color flow cytometry. PBMCs were incubated with the next surface-labeled monoclonal-antibodies, CD3-PercP, CCR7-PECY7 (Becton-Dickinson, BD, CA, USA), CD8-Alexa405, CD45RA-APC (Caltag, Carlsbad, CA, USA) and CD27-APCAlexa780 (eBioscience, San Diego, CA, USA).

For intracytoplasmic staining, cells were fixed and permeabilized (Fix and Perm, Caltag, Carlsbad, CA, USA), and cytokines were stained with IL-4-PE, IFNγ-Alexa700 and IL-17A-FITC (Becton-Dickinson, BD, CA, USA). All samples were stained with a dead cell-discriminator simultaneously with antibody addition (Fixable aqua dead cell stain kit for 405 nm excitation; Molecular Probes, Eugene, OR, USA). 

Samples were acquired in a FacsAria-II flow cytometer and were analyzed using FacsDiva 5.0 and Flow-Jo 7.0 software (Becton-Dickinson, BD, San Jose, CA, USA).

### 4.7. Cytokines Serum Levels

Samples were obtained in sterile clotting tubes from peripheral blood by ante-cubital venipuncture. These samples were centrifuged at 863× *g* for 20 min at 4 °C, aliquoted, identified and labeled, and frozen at –80 °C. Then, they were thawed and evaluated using the high sensitivity human MILLIPLEX^®^ kit to simultaneously measure IFNγ, IL-4 and IL-17A (Millipore) following the manufacturer’s instructions and revealing the results by Luminex (MAGPIX^®^ system). The tested cytokines had the following sensitivity limits (0.48 pg/mL for IFNγ, 1.12 pg/mL for IL-4 and 0.33 pg/mL for IL-17A). The results were analyzed using Analyst 5.1 software MILLIPLEX^®^ (MerkMillipore, Boston, MA, USA).

### 4.8. STATs Flow Cytometry 

The spontaneous and stimulated CD4^+^T-lymphocyte subset expression of STAT-1, STAT-3 and STAT-6 was assessed by in vitro phosphoprotein intracytoplasmic staining. To evaluate STAT-1, STAT-3 and STAT-6 phosphorylation, PBMCs were stimulated for 15′ with 40.000 U/mL of IFNα for STAT-1, 0.1μg/mL of IL-6 for STAT-3 and 0.1 μg/mL of IL-4 for STAT-6. Additionally, the total STAT-1, STAT-3 and STAT-6 protein was analyzed in CD4^+^T-lymphocyte subsets.

Surface and intracellular lymphocyte staining. For intracytoplasmic STAT staining, PBMCs were fixed, permed (BDPhosphoflow, Becton-Dickinson, BD, San Jose, CA, USA) and stained with the subsequent surface-labeled monoclonal-antibodies CD4-FITC, CD27-PE, CD3-Percp (Becton-Dickinson, BD, San Jose, CA, USA) CD45RA-Alexa405 (eBioscience, San Diego, CA, USA) and with the STAT1-FITC, STAT3-Pacificblue or STAT6-PE (Becton-Dickinson, BD, San Jose, CA, USA) intracellular-labeled monoclonal-antibodies against phosphorylated or total STAT protein.

The quality control of the flow cytometer was performed daily according to the manufacturer’s instructions (Becton-Dickinson, BD, San Jose, CA, USA). The staining protocol and quality and analysis controls were performed by ‘fluorescence minus one control’ as described by Roederer et al. [49], and the flow cytometry results were presented following the guidelines of the International Society of Advancement of Cytometry (ISAC) [50].

### 4.9. Statistical Analysis

Analyses were performed using SPSS-22 software (SPSS-IBM, Armonk, NY, USA). Since most variables did not fulfill the normality hypothesis, the Mann–Whitney U-test for nonparametric data was used to analyze differences between groups, and analysis of variance followed by Wilcoxon tests were used for within group analyses. The significance level was set at *p* < 0.05.

## 5. Conclusions

The results revealed a heterogeneous function of CD4+ T-lymphocytes subsets in the early clinical stages of RA with potential pathogenic relevance:

The numbers of circulating Th1, Th2 or Th17 CD4^+^T-lymphocytes subsets in the early clinical stages of RA show two different profiles of cytokine producing CD4^+^ T-lymphocyte subsets associated to a response or not associated to the MTX treatment of the RA patients.

The pattern of IL-17^+^, IFNγ^+^ and IL-4^+^ CD4^+^ T production detected in new-onset DMARD-naïve RA patients could be modified by MTX treatment.

Results may offer a way of identifying which patients will respond, or not, to MTX treatment.

## Figures and Tables

**Table 1:** Patient demographics and clinical and biological characteristics at baseline.

	Healthy Controls (*n* = 29)	Eventual Responders (*n* = 31)	Eventual Non-Responders (*n* = 16)	
Variables	(mean ± SD)	(mean ± SD)	(mean ± SD)	*p*-value
Age (years)	48.70 ± 12.01	51.60 ± 10.01	52.02 ± 9.48	0.823
Gender (women)	72.10%	74.19%	75.00%	0.902
CRP (mg/L)	-	16.12 ± 6.39	16.57 ± 5.33	0.942
Rheumatoid factor (+) Prevalence (+)	-	225.34 ± 88.46 87.09%	233 ± 91.18 87.50%	0.907 0.841
Anti-CCP (IU/mL) Prevalence (+)	-	435.72 ± 358.15 77.41%	431.08 ± 276.10 81.25%	0.965 0.809
DAS28	-	3.62 ± 0.49	3.69 ± 0.46	0.689
Erosions (+)	-	27.01%	27.53%	0.759
HAQ	-	0.76 ± 0.56	0.78 ± 0.79	0.844

CRP, C-reactive protein; anti-CCP, anticyclic citrullinated peptide antibody; DAS28, Disease Activity Score 28; HAQ, Health Assessment Questionnaire.

## Abbreviations

IFNg	Interferon gamma
IL-4	Interleukin-4
IL-17	Interleukin-17a
RA	Rheumatoid arthritis
DMARDs	Disease-Modifying antirheumatic drugs
PBMC	Peripheral blood mononuclear cells
FITC	Fluorescein-Isothiocyanate
PE	Phycoerythrin
Apc-Alexa780	Allophycocyanin-alexa-780
Pe-Cy7	Phycoerythrin-Cyanine Seven
mAb	Monoclonal antibodies
MTX	Methrotexate
Th	T helper
T_N_	T naïve
T_CM_	T central memory
T_EM_	T effector memory
T_E_	T effector
PMA	Phorbol-12-Myristate-13-Acetate
